# Prevalence of Methicillin-Resistant *Staphylococcus* sp. (MRS) in Different Companion Animals and Determination of Risk Factors for Colonization with MRS

**DOI:** 10.3390/antibiotics8020036

**Published:** 2019-04-05

**Authors:** Igor Loncaric, Alexander Tichy, Silvia Handler, Michael P. Szostak, Mareike Tickert, Magda Diab-Elschahawi, Joachim Spergser, Frank Künzel

**Affiliations:** 1 Institute of Microbiology, University of Veterinary Medicine, 1210 Vienna, Austria; SilviaHandler@gmx.at (S.H.); michael.szostak@vetmeduni.ac.at (M.P.S.); Joachim.spergser@vetmeduni.ac.at (J.S.); 2 Department of Biomedical Sciences, University of Veterinary Medicine, 1210 Vienna, Austria; alexander.tichy@vetmeduni.ac.at; 3 FH Campus Wien, 1100 Wien, Austria; 4 Clinical Unit of Internal Medicine Small Animals, University of Veterinary Medicine, 1210 Vienna, Austria; info@kleintierpraxis-west.de (M.T.); frank.kuenzel@vetmeduni.ac.at (F.K.); 5 Division for Hospital Hygiene, Clinical Institute for Hygiene and Medical Microbiology, Vienna General Hospital, Medical University of Vienna, 1090 Vienna, Austria; magda.diab-elschahawi@meduniwien.ac.at

**Keywords:** MRSA, methicillin resistance, animals, antibiotic resistance, SCC*mec* typing, *dru* typing, risk factors

## Abstract

The aim of this study was to detect the prevalence of methicillin-resistant *Staphylococcus* sp. (MRS) in populations of companion animals that either have previously been exposed or have not been exposed to antibiotic therapy or veterinary facilities, and if owners’ healthcare profession had an influence on colonization with MRS. In addition, the antimicrobial resistance pheno- and genotype were investigated and risks for colonization with MRS were assessed. During this study, 347 nasal swabs (dogs *n* = 152; cats *n* = 107; rabbits *n* = 88) were investigated for the presence of methicillin-resistant *Staphylococcus aureus* (MRSA). In addition, 131 nasal swabs (dogs *n* = 79; cats *n* = 47; rabbits = 3; guinea pigs = 2) were examined for the presence of MRSA but also other MRS. In total, 23 MRS isolates belonged to nine staphylococcal species: *Staphylococcus epidermidis* (*n* = 11), *Staphylococcus warneri* (*n* = 3), *Staphylococcus hominis* (*n* = 2), *Staphylococcus pseudintermedius* (*n* = 2), and singletons *Staphylococcus cohnii*, *Staphylococcus sciuri*, *Staphylococcus fleurettii*, *Staphylococcus lentus*, and *Staphylococcus haemolyticus*. Twenty isolates displayed a multidrug-resistant phenotype. Various resistance and biocide resistance genes were detected among the examined staphylococci. Risk assessment for MRS colonization was conducted using a number of factors, including animal species, breed, age, gender, recent veterinary health care hospitalization, and antibiotic prescription, resulting in recent veterinary health care hospitalization being a significant risk factor. The detection of multidrug-resistant MRS in healthy animals is of importance due to their zoonotic potential.

## 1. Introduction

Members of the genus *Staphylococcus* (*S*.) are part of the normal skin flora of animals and humans. Staphylococci are important pathogens with a wide host range and are capable of causing serious infections of the skin and many other tissues [1,2].

Antimicrobial resistance among staphylococci is based on a wide variety of resistance genes. The most important is methicillin resistance mediated mainly by the *mecA* gene, which encodes for a penicillin-binding protein (PBP), PBP-2a [3]. Methicillin-resistant staphylococci (MRS) are important pathogens in human and veterinary medicine and are often multidrug resistant, extremely limiting therapeutic options. MRS are recognized as one of the most important risks for human and animal health [1,4]. In addition to antibiotic therapy, antibacterial disinfectants, such as those based on chlorhexidine gluconate and quaternary ammonium compounds (QACs), used as topical antibacterial therapy can provide an alternative treatment option for staphylococcal superficial infections and therefore limit the need for oral antibiotics [1,5]. On the other hand, there is a growing concern about the emergence of disinfectant-resistant staphylococci [6]. Reduced susceptibility to chlorhexidine gluconate and QACs is usually mediated by energy-dependent multidrug efflux proteins, which show increased expression in response to selective pressure from disinfectant use [7]. Plasmid-encoded efflux pump genes, such as *qacA/B* and *qacC* (*smr*), confer tolerance to both chlorhexidine gluconate and QACs [7]. The reported prevalence of MRS is higher in animals exposed to veterinary healthcare units and antimicrobial therapy [8,9,10], suggesting that these are risk factors for colonization. Despite high proximity to humans, the importance of companion animals as reservoirs of human infections is still poorly understood.

Therefore, the aims of the present study were to: (i) detect the prevalence of MRS in a different population of small animals, including animals exposed or not to antibiotic therapy and/or veterinary and/or human healthcare facilities; (ii) investigate the antimicrobial resistance pheno- and genotype; and (iii) determine risks for colonization with MRS.

## 2. Results

Within the first part of the study, which dealt solely with methicillin-resistant *S. aureus* (MRSA) colonization, MRSA could not be detected in any of the examined animals and, therefore, this part of the present study was not included in statistical analyses. During the second part of the present study, which dealt with all MRS (including *S. aureus*), nasal colonization with MRS was detected in 23 animals. Also, nasal colonization with MRSA could not be detected during the second part. Based on *rpoB* DNA sequencing, the predominant staphylococcal species was *S. epidermidis* (*n* = 11), followed by *S. warneri* (*n* = 3), *S. hominis* and *S. pseudintermedius* (each *n* = 2), and singletons of *S. cohnii*, *S. fleurettii*, *S. haemolyticus*, *S. lentus*, and *S. sciuri* (Table 1). Nucleotide accession numbers are MK648115–MK648137.

All isolates were penicillin-, cefoxitin-, or oxacillin- (in the case of *S. pseudintermedius*) resistant, *mecC* negative but *mecA* positive, and all but one isolate carried the *blaZ* gene. Among tested isolates, phenotypic resistance to erythromycin (*n* = 20) was most frequently detected followed by resistance to trimethoprim-sulfamethoxazole (*n* = 11). *erm*(C) (*n* = 9) and *dfrA* (*n* = 13) were the most common resistance genes among tested isolates. Nine isolates were resistant to ciprofloxacin. Twenty isolates displayed a multidrug-resistant phenotype (an isolate that is not susceptible to at least one agent in at least three antimicrobial classes) [11] (Table 1). Beside the frequently detected *erm*(C) and *dfrA* resistance genes, the following resistance genes were infrequently detected among MRS: tetracycline resistance gene: *tet*(K), aminoglycoside resistance genes: *aacA-aphD* and *aac(6′)-Ie*, macrolides, lincosamides, and streptogramin B resistance gene: *erm*(B), macrolides and streptogramin B resistance gene: *msr*(A), trimethoprim resistance gene: *dfrG*, nonfluorinated phenicols resistance gene: *cat*_pC221_, and fusidic acid resistance gene: *fusC*. Eight isolates harboured the *qacA/B* gene and five *smr* genes. The presence of both *qac* genes was detected in two isolates, one *S. epidermidis* and one *S. warneri*. SCC*mec* typing revealed that SCC*mec* type IV was predominant (*n* = 7). SCC*mec* type I was detected in two *S. hominis* isolates, whereas two *S. pseudintermedius* isolates carried SCC*mec* II–III. Two singletons, SCC*mec* II and V, were detected in *S. sciuri* and *S. lentus*, respectively. In nine isolates, SCC*mec* could not be assigned. *dru* type dt10a was most predominant (*n* = 10), whereas dt9a, dt11a, dt8b, dt11c, and dt7ah were also detected among tested isolates. Three isolates were non-typeable. All pheno- and genotyping results are summarized in Table 1. The Panton–Valentine leukocidin (PVL) gene, genes of staphylococcal enterotoxins (SE), and toxic shock syndrome toxin (TSST) could not be detected.

Regarding risk factors, only recent veterinary health care hospitalization differed significantly (*p* = 0.046) between animals with and without MRS colonization. Animals receiving antibiotic therapy had a 2.5 times higher chance to become colonized with MRS, but the value is not significant. In addition, all other evaluated risk factors did not differ significantly (Table 2).

## 3. Discussion

There are more than 2 million companion animals in Austria, but information regarding MRS other than MRSA isolated from these animals is practically nonexistent, except for one study [12]. To date, in Austria MRS could only be detected in companion animals of clinical facilities [13,14]. In the present study, several different MRS but not MRSA were isolated from different host species. Several studies have reported a low prevalence regarding carriage of *S. aureus*, *S. pseudintermedius*, and *S. schleiferi* in dogs and cats. These studies were summarized in Morris et al. [1]. A prevalence between 0.0% and 6% was detected in other studies with its highest observed in a study from the United States in pets of MRSA-infected owners [1]. Interestingly, a low presence of MRSA has also been observed among clinical specimens derived from companion animals in Austria [13]. Since the prevalence of carriage of methicillin-resistant *S. pseudintermedius* (MRSP) varies greatly (0.0 to 45%) in different studies, the prevalence of 1.5% observed during the present study is rather low [1].

Comparatively to methicillin-resistant coagulase-positive staphylococci, information on the prevalence of methicillin-resistant coagulase-negative staphylococci (MRCoNS) in companion animals is scarce. Most of the MRS isolated in the present study belonged to the species *S. epidermidis*. *S. epidermidis* is the most reported nosocomial pathogen among MRCoNS in human medicine [15], and has often been detected in companion animals either as a primary pathogen [16] or isolated from healthy animals [17,18,19]. *S. warneri* is considered to be a common commensal and its methicillin-resistant (MR) variant has been rarely isolated from clinical sites [15] or as a colonizer [17,20]. During the present study, two canine isolates of MR *S. hominis* could be detected. *S. hominis* is second to *S. epidermidis* in causing infections in human medicine [2], and its MR variant has already been reported to be associated with infection in companion animals [15].

However, reports on the carriage of MR *S. hominis* in these animals are scarce. The other five staphylococcal isolates detected during this study were singletons (*S. sciuri*, *S. fleurettii*, *S. lentus*, *S. hamolyticus*, and *S. cohnii*). Three of the six belonged to the *S. sciuri* group (*S. sciuri*, *S. fleurettii*, and *S. lentus*). Members of the *S. sciuri* group are commensal animal-associated staphylococci. Species of this group are known to carry different homologues of the methicillin resistance gene *mecA* in their chromosomal DNA but also the *mecA* gene that is located on an SCC*mec* [21]. The prevalence of MR members of *S. sciuri* group is low (usually lower than 1%), as described in studies dealing with the carriage of MRS from companion animals [17,19]. Comparable prevalence rates were observed for MR *S. haemolyticus* [22]. MR *S. haemolyticus* has also been detected in infections of companion animals, albeit rarely [16]. Little is known about MR *S. cohnii*, which was isolated from a single swab during the present study, and its role as a causative agent of animal infection because it has only occasionally been isolated from animal infection [16,23].

The multidrug-resistant pheno- and genotypes observed in the majority of MRS examined during this study are in concordance with studies that examined the diversity of MRSP and MRCoNS isolated from companion animals [16,24]. The detection of staphylococci resistant to β-lactams combined with a high resistance frequency to fluoroquinolones, which are the most commonly used antibiotics in veterinary practices, represents important information. These two classes of antibiotics have been shown to represent a significant risk factor for the selection of MRSA [25] and, therefore, similar effects to other MRS are to be anticipated. Thus, MRS isolates additionally resistant to fluoroquinolones should be viewed with caution.

As for MRS carriage, relatively little is known regarding the resistance of MRS to antiseptics. In staphylococci, resistance to biocides, such as antibacterial disinfectants based on chlorhexidine gluconate and QACs, is efflux-mediated resistance conferred by the *qac* genes, which seem to be the most widespread [26]. In the present study, the determination of the biocide resistance genes *qacA/B* and *smr* was performed. These genes were detected among *S. epidermidis*, *S. warneri*, and *S. hominis*, which may suggest an association with resistance to antiseptics. To the best of the authors’ knowledge, there are no studies describing the detection of *qac* genes in MRS, especially MRCoNS, associated with MRS colonization of companion animals. A comparable study [27] reported the detection of biocide-resistance genes among equine MRS. In that study, *qac* genes were detected in *S. cohnii* (*qacB*, *qacG*-like), *S. haemolyticus* (*qacA*, *sh-fabI*), and three other MRSA strains (*qacB*, *qacA*, *sh-fabI*). Information about *S. epidermidis*, *S. warneri*, and *S. hominis* of animal origin carrying *qacA/B* and *smr* genes is scarce. In a study on disinfectant resistance genes among bovine and caprine staphylococci from Norway, *qacA/B* was detected in bovine *S. epidermidis* as well as in bovine and ovine *S. warneri*. The *smr* gene was detected in caprine *S. warneri* as well as bovine *S. epidermidis* and *S. hominis* [28]. Our study had the limitation that biocide susceptibility testing was not performed. Therefore, it was not possible to associate the detection of *qac* genes with the increase in minimum bactericidal concentration (MBC) values. In addition, studies that dealt with the presence of *qac* genes, and the determination of minimum bactericidal concentrations in staphylococci of animal origin, observed no association between detection of *qacA/B* and *smr* genes and higher MBC values in comparison to *qac*-negative strains [27,28].

Even though previous antimicrobial therapy is recognized to be a risk factor for the development of infection/colonization with MRS [29,30], the difference between colonized and non-colonized animals was not significant. However, animals receiving antibiotic therapy in the present study had a 2.5 times higher chance to become colonized, and the prior hospitalization of animals was determined to be a risk factor for MRS colonization in [29,30], which is in a concordance with the result obtained during the present study.

## 4. Materials and Methods

The study was discussed and approved by the institutional ethics and animal welfare committee in accordance with Good Scientific Practice (GSP) guidelines and national legislation.

To determine the presence of nasal colonization of MRSA, the study was divided into two parts: (1) 347 nasal swabs (dogs *n* = 152; cats *n* = 107; rabbits *n* = 88) were investigated for the presence of methicillin-resistant *S. aureus* (MRSA) solely; and (2) 181 nasal swabs (dogs *n* = 107; cats *n* = 47; rabbits = 3; guinea pigs = 2) were examined for the presence of MRSA but also other MRS (Table S1). Swabs for cultivation experiments were transported from the veterinary clinic to the laboratory within 2 h of collection and processed in the laboratory within 4 h.

Isolation of MRS was performed as previously described [31,32]. Methicillin resistance was confirmed by agar disk diffusion as well as PCRs for *mecA* and *mecC* [32]. Antimicrobial susceptibility testing was performed by agar disk diffusion according to CLSI standards [33] for the following antimicrobial agents (µg/disk): cefoxitin (30), oxacillin (1), ciprofloxacin (5), amikacin (30), gentamicin (10), tetracycline (30), erythromycin (15), clindamycin (2), chloramphenicol (30), trimethoprim-sulfamethoxazole (1.25 + 23.75), nitrofurantoin (300), rifampicin (5), and linezolid (30) (Beckton Dickinson (BD); Heidelberg, Germany). *S. aureus* ATCC^®^ 29213 was used as quality control. Isolates were subjected to SCC*mec* typing as described previously [31]. Isolates were further subjected to *dru* typing [34]. Detection of antibiotic resistance genes was performed by PCR. PCR was performed for detecting the presence of the following antibiotic resistance genes: *blaZ* (confers resistance to penicillins except for isoxazolyl-penicillins) [35]; *erm*(A), *erm*(B), and *erm*(C) (confer resistance to macrolides, lincosamides, and streptogramin B) [13,36]; *msr*(A) (confers resistance to macrolides and streptogramin B) [13]; *cfr* (confers resistance to all phenicols, lincosamides, oxazolidinones, pleuromutilins, and streptogramin A) [36]; *fexA* (confers resistance to all phenicols) [37]; *cat*_pC194_, *cat*_pC221_, and *cat*_pC223_ (confer resistance to nonfluorinated phenicols and chloramphenicol) [38]; *aac-aphD* (confers resistance to the aminoglycosides gentamicin, kanamycin, tobramycin, and, when overexpressed, to amikacin) [13]; *ant(6′)-Ia* and *str* (confer resistance to the aminoglycoside streptomycin) [39]; *dfrA*, *dfrD*, *dfrG*, and *dfrK* (confer resistance to trimethoprim) [13,40]; *tet*(K) and *tet*(L) (confer resistance to tetracyclines, except for minocycline and glycylcyclines) [13,41,42]; *tet*(M) (confers resistance to tetracyclines, including minocycline but excluding glycylcyclines) [13]; and *tet*(O) [40]. In the case of *S. pseudintermedius*, the presence of SCC*mec* II–III was demonstrated by multiplex PCR as described elsewhere [24]. PCRs targeting the *qacA/B* (confers high-level resistance to antiseptics) and *smr* (confers low-level resistance to antiseptics) genes were performed as previously described [26]. PCRs targeting Panton–Valentine leukocidin (PVL) genes and the detection of staphylococcal enterotoxins (SE) and toxic shock syndrome toxin (TSST) were performed as previously described [13]. All PCRs within the present study were performed using the standard protocols. Isolates were identified to the species level by *rpoB* sequencing [43]. All *rpoB* DNA sequences were deposited into GenBank.

In order to determine risk factors during the second part of the present study, differences between species (dog, cat, rabbit, guinea pig), breed, age, gender, husbandry conditions (indoor/outdoor), recent veterinary health care hospitalization (during the last 6 months), and pretreatment with antimicrobial substances (during the last 6 months) for colonization with MRS were analysed using a Chi-square test. In addition, statistical analysis included whether there was close contact between animal and owner and whether the owner was a health care professional. In order to evaluate whether the origin and health status of animals might be risk factors for colonization, three groups were generated: Group 1 (clinically healthy animals from employees of the University of Veterinary Medicine Vienna), Group 2 (clinically healthy animals from owners other than employees of the University of Veterinary Medicine Vienna), and Group 3 (animals that were presented as patients at the University of Veterinary Medicine Vienna). A value of 5% (*p* < 0.05) was considered significant for all statistical analyses. Calculations were performed using IBM SPSS v24^®^ (IBM Corp., Armonck, NY, USA).

## 5. Conclusions

In conclusion, the present study is the first dealing with the carriage of different MRS from companion animals in Austria. Due to high proximity of companion animals to humans, the detection of MRS in healthy animals is of importance, since the transmission of MRS strains between companion animals and humans may occur and, therefore, requires further prudent use of antibiotics in veterinary settings.

## Figures and Tables

**Table 1:** Molecular characterization and antimicrobial resistance profile of the investigated methicillin-resistant *Staphylococcus* sp. isolates.

ID	Species	SCC*mec*	*dru* Type	Non β-lactam Phenotype **	Non b-lactam Resistant Genes	QAC Genes ***
A 36	*S. epidermidis*	nt *	dt10a	CIP, AMK, GEN, ERY, CLI	*dfrA*, *erm*(C)	*qacA/B*
A 39	*S. epidermidis*	IV	dt10a	CIP, TET, ERY, CLI	*dfrA*, *erm*(C)	*qacA/B*
A 40	*S. epidermidis*	IV	dt10a	TET, ERY, CLI	*dfrA*, *erm*(C), *tet*(K)	
A 41	*S. pseudintermedius*	II–III	dt9a	CIP, AMK, GEN, ERY, CLI, SXT	*aacA-aphD*, *erm*(B), *dfrG*	
A 50	*S. epidermidis*	nt	dt11a	AMK, GEN, TET, ERY, SXT	*msr*(A), *aacA-aphD*, *tet*(K) *drfG*, *aac(6′)-Ie*	*smr*
A 57	*S. sciuri*	II	dt8b	AMK, GEN, ERY	*dfrA*, *msr*(A), *aacA-aph*, *aac(6′)-Ie*	
A 63	*S. warneri*	IV	dt10a	ERY, SXT	*dfrA*, *msr*(A)	
A 68	*S. epidermidis*	nt	nt		*dfrA*, *aacA-aphD*, *erm*(C)	*qacA/B*
A 72	*S. pseudintermedius*	II–III	dt9a	CIP, AMK, GEN, TET, ERY, CLI, SXT	*aacA-aphD*, *tet*(K), *erm*(B), *dfrG*, *aac(6′)-Ie*	
A 73	*S. warneri*	IV	dt10a	ERY	*msr*(A)	*smr*
A 112	*S. epidermidis*	IV	dt10a		*dfrA*	
A 127	*S. warneri*	IV	dt10a	ERY	*dfrA*, *msr*(A)	*qacA/B*, *smr*
A 141	*S. cohnii*	nt	dt11a	ERY, SXT	*msr*(A)	
B 7	*S. lentus*	V	dt10a	CIP, GEN, TET, ERY, SXT	*aacA-aphD*, *tet*(K), *aac(6′)-Ie*	
B 23	*S. epidermidis*	nt	nt	CIP, ERY, CLI, SXT	*dfrA*, *erm*(C)	
B 25	*S. epidermidis*	IV	dt8b	ERY, CLI	*dfrA*, *erm*(C)	*qacA/B*
B 27	*S. epidermidis*	nt	dt8b	CIP, TET, ERY, CLI	*dfrA*, *msr*(A), *tet*(K)	
B 37	*S. epidermidis*	nt	dt10a	CIP, AMK, GEN, TET, ERY, CLI, SXT	*dfrA*, *aacA-aphD*, *erm*(C), *aac(6′)-Ie*	*qacA/B*, *smr*
B 49	*S. fleurettii*	nt	nt	TET, CHL	*tet*(K), *cat*_pC221_	
B 50	*S. haemolyticus*	nt	dt11c	CIP, AMK, GEN, ERY, CLI, SXT	*msr*(A), *aacA-aphD*, *erm*(C), *aac(6′)-Ie*	
C 10	*S. epidermidis*	nt	dt10a	ERY, CHL, SXT	*dfrA*, *cat*_pC221_	*qacA/B*
162	*S. hominis*	I	dt7ah	ERY, CLI, CHL, SXT	*erm*(C), *fusC*, *cat*_pC221_	*smr*
166	*S. hominis*	I	dt9a	ERY	*msr*(A)	*qacA/B*

* non-typeable. ** CIP = ciprofloxacin; AMK = amikacin; GEN = gentamicin; TET = tetracycline; CHL = chloramphenicol; ERY = erythromycin; CLI = clindamycin; SXT = trimethoprim-sulfamethoxazole. *** Quaternary Ammonium Compounds resistance genes.

**Table 2:** Statistical analysis of risk factors.

Risk Factor	*p* Value
Species (dog, cat, rabbit, guinea pig)	0.664
Breed	0.833
Age	0.182
Gender	0.06
Husbandry conditions (indoor/outdoor)	0.502
Recent veterinary health care hospitalization (during the last 6 months)	0.046
Pretreatment with antimicrobial substances (during the last 6 months)	0.096
Close contact	0.2
Owner’s health care profession	0.223
Origin and health status of animals	0.993

## Supplementary Materials

The following are available online at https://www.mdpi.com/2079-6382/8/2/36/s1. Table S1: detailed information on examined animals: * MRS: methicillin-resistant staphylococci, MRSA: methicillin-resistant *S. aureus*; ** m = male, w = female, mk = male castrated, wk = female castrated; *** yes = indoor, no = outdoor; NA = not available; bold/italic = positive samples.
